# Urinary Glyphosate Exposure and Risk of Obstructive Airway Diseases in Youth and Adults: Population-Based Evidence from U.S. Biomonitoring Data

**DOI:** 10.3390/ijerph23040442

**Published:** 2026-03-31

**Authors:** Hiba Farooq Siddiqui, Sundus Farooq Siddiqui, Marrita Rabadi, Anfal Mohamed Shalaby, Abdullatif Ibrahim Al-Hor, Maryam A. Al-Khulaifi, Humam Emad Rajha, Giridhara Rathnaiah Babu, Angham Ibrahim Tartour

**Affiliations:** 1 College of Medicine, QU Health, Qatar University, Doha 2713, Qatar; hs2209485@qu.edu.qa (H.F.S.); ss2209486@qu.edu.qa (S.F.S.); mr2204570@qu.edu.qa (M.R.); aa2206612@qu.edu.qa (A.M.S.); aa2002171@qu.edu.qa (A.I.A.-H.); ma2204227@qu.edu.qa (M.A.A.-K.); hr2003606@qu.edu.qa (H.E.R.); 2 Department of Population Medicine, College of Medicine, QU Health, Qatar University, Doha 2713, Qatar; aa1304397@qu.edu.qa

**Keywords:** National Health and Nutrition Examination Survey (NHANES), glyphosate, COPD, asthma, airway disease, herbicide

## Abstract

**Highlights:**

**Public health relevance—How does this work relate to a public health issue?**

- Glyphosate exposure is widespread and may affect respiratory health in the general population.
- Obstructive airway diseases such as asthma and COPD contribute substantially to global morbidity.

**Public health significance—Why is this work of significance to public health?**

- Increased glyphosate exposure correlates with asthma attacks and emergency visits.
- Findings contribute evidence on environmental determinants of asthma and COPD in youths and adults.

**Public health implications—What are the key implications or messages for practitioners, policy makers and/or researchers in public health?**

- Results highlight the need for monitoring and prevention strategies to reduce glyphosate exposure.
- Longitudinal studies are needed to clarify causal links between herbicide exposure and respiratory disease.

**Abstract:**

Glyphosate herbicide has been linked with airway diseases in adults. Evidence remains inconsistent, without investigations conducted in youthful populations. This study aims to examine the association between glyphosate and obstructive airway diseases in adult and youth samples in the National Health and Nutrition Examination Survey. Glyphosate exposure was categorized into tertiles. Separate multiple logistic regressions were conducted for adults and youths, adjusting for relevant covariates. Analyses were stratified by age. Sensitivity analyses explored associations with past-year asthma-related events. In total, 4031 adults and 1692 youths were included. There was a significant dose–response relationship between glyphosate and airway diseases in adults (tertile 2: aOR = 1.17, 95% UI: 0.94, 1.46, *p* = 0.15; tertile 3: aOR = 1.29, 95% UI: 1.02, 1.62, *p* = 0.03). Weak associations were shown between glyphosate and asthma in the youth sample (aOR = 1.08, 95% UI: 0.74, 1.58, *p* = 0.69); however, the relationship was more pronounced in adolescents (aOR = 1.33, 95% UI: 0.82, 2.15, *p* = 0.24). A dose–response pattern was observed between recent exposure and temporal outcomes in adults, reaching up to 75% higher odds of past-year asthma attacks among participants with glyphosate levels at the third tertile (tertile 3: aOR = 1.75, 95% UI: 0.92, 3.36, *p* = 0.09). These warrant timely monitoring and prevention strategies, with future research focusing on investigating longitudinal associations.

## 1. Introduction

Glyphosate (N-phosphonomethyl glycine) is a broad-spectrum herbicide and the most used weed killer in the U.S. and globally [1]. Its application surged after the introduction of glyphosate-resistant genetically modified crops, resulting in a tenfold increase within the first three decades [2]. Glyphosate-based products are applied across agriculture, forestry, and residential settings [1], contributing to their environmental presence in air, water, soil, and food [3,4].

Although glyphosate does not bioaccumulate and exhibits low soil mobility, it has the potential to become airborne during and after application, increasing the risk of exposure [5,6]. The U.S. Centers for Disease Control and Prevention (CDC) has reported extensive glyphosate exposure among Americans through the National Health and Nutrition Examination Survey (NHANES). In the 2013–2014 NHANES cycle, glyphosate was found in approximately 81% of participants’ urine [7], with similar rates in subsequent cycles, reaching children as young as 3 years of age.

The potential impact of glyphosate on human health has emerged as a significant area of concern, particularly due to its association with chronic health effects rather than acute toxicity [8,9,10]. Evidence suggests that high-level glyphosate exposure, particularly through inhalation or dermal absorption during spraying, may trigger airway inflammation [11,12,13]. Previous research has linked pesticide/herbicide exposure to respiratory outcomes [14,15,16]. However, few studies have specifically examined glyphosate, and most were limited to occupational cohorts, particularly farming populations.

Findings are inconsistent regarding the association between glyphosate exposure and airway diseases. One study investigated the association between urinary glyphosate tertiles and nine health outcomes in the U.S., including chronic obstructive pulmonary disease (COPD) [8]. This study found no associations between glyphosate and COPD. Another recent U.S. study reported strong associations between urinary glyphosate concentrations and COPD among adults aged ≥40 years [15]. Nonetheless, the study did not examine asthma, excluded younger populations, including children, and lacked adjustment for family history of obstructive airway disease, raising concerns about residual confounding [15].

This study examined the association between urinary glyphosate concentrations and obstructive airway diseases, including asthma and COPD, in both adult and youth (asthma) samples from NHANES, while accounting for key sociodemographic, lifestyle, and clinical covariates.

## 2. Methods

### 2.1. Study Design and Sample

This was a cross-sectional study utilizing data from three NHANES cycles, covering the years 2013–2014, 2015–2016, and 2017–2018.

NHANES uses a combination of in-person and telephone interviews, physical examinations, and laboratory analyses from a sample of approximately 5000 people in the U.S. each year [17]. The representation of diverse demographics is ensured through a multistage probability sampling technique across all 50 states. The NHANES study protocol has received approval from the Institutional Review Board of the National Center for Health Statistics (NCHS), guaranteeing ethical oversight. Informed written consent was obtained from all participants. In cases involving participants under the age of 18, consent was provided by parents or guardians. The authors had no access to information that could identify individual participants.

The initial NHANES sample comprised 29,400 participants of all ages recruited from 2013 to 2018. Adult participants (aged ≥20 years) were included if they had available data on urinary glyphosate levels and either asthma, COPD, chronic bronchitis, or emphysema. Youth participants (aged <20 according to NHANES) were included if they had available data on urinary glyphosate levels and asthma status. Pregnant individuals (identified through self-reported questionnaires or laboratory results of the urinary pregnancy test) were excluded. Those with missing covariates were excluded.

### 2.2. Exposure Measure: Urinary Glyphosate Level

The current study did not directly retrieve or analyze urine samples. NHANES collects biospecimens on a continuous basis and releases the laboratory data in 2-year cycles. Urine glyphosate measurements used in this study were obtained from the publicly available NHANES laboratory datasets for the relevant cycles. NHANES does not provide exact collection dates for individual samples. Data on urine glyphosate was accessed for this study on 26 February 2025.

In NHANES, each participant provides a single spot urine sample (200 μL) during the Mobile Examination Center (MEC) visit; samples are aliquoted on-site, frozen, and shipped to the CDC laboratory (CDC, Atlanta, GA, USA) for analysis using 2-dimensional ion chromatography–tandem mass spectrometry (IC-MS/MS) with isotope dilution, a validated and standardized method. Participants aged 3–5 years and a one-third subsample aged ≥6 years provided urine samples in each cycle [18]. All analyses followed Clinical Laboratory Improvement Amendments (CLIA) quality standards [19], with concentrations reported in nanograms per milliliter (ng/mL).

### 2.3. Outcome Measure: Obstructive Airway Diseases

COPD status was determined based on the response to whether the participant “had ever been told they had COPD”. Those who reported having emphysema and chronic bronchitis were also classified as having COPD. Emphysema status was determined based on responses to questions regarding whether the participant was ever told they had emphysema, the age at diagnosis, or whether they were taking emphysema prescription medications. Chronic bronchitis status was determined based on responses regarding whether the participant had ever been diagnosed with chronic bronchitis, the age of diagnosis, whether they still had chronic bronchitis at the time of the survey, and the use of prescription medications.

Asthma status was determined based on the answers to the following: whether the participant “had ever been diagnosed with asthma,” “had an asthma attack in the past year,” “had an emergency care visit for asthma in the past year,” and the age of asthma diagnosis. Additionally, participants who used asthma prescription medications were classified as having asthma.

Questionnaire data were used to classify the outcomes of interest, as NHANES did not report spirometry lung function data for the relevant years (2013–2018) that reported data on glyphosate.

### 2.4. Covariates

Covariates included age, sex, race (Asian, White, Black, Other/multiracial), socioeconomic status (income-to-poverty ratio < 1 or ≥1), BMI, smoking, and employment. Active smoking was defined by current smoking, >100 lifetime cigarettes, or serum cotinine ≥ 10 ng/mL. Secondhand exposure was defined as smoke exposure within the past 7 days at home, work, vehicles, or other indoor spaces. Family history of asthma was included for asthma models. Employment was defined as currently having a job or business; workplace smoke exposure was also considered.

### 2.5. Statistical Analysis

Descriptive statistics summarized demographic and clinical characteristics. Glyphosate exposure was divided into tertiles to assess dose–response relationships. Multivariable logistic regression estimated associations between urinary glyphosate and obstructive airway diseases, with separate models for asthma and COPD. A composite adult outcome (“any obstructive airway disease”) included those with asthma and/or COPD.

Variables potentially associated with both exposure and outcome were identified through a comprehensive literature review and subject expert consultations. These variables were incorporated into a directed acyclic graph (DAG), enabling the identification of a minimal set of covariates necessary to control for confounding. Additionally, prognostic variables for asthma were adjusted to mitigate the potential magnification of risk factors [20]. This approach is regarded as the most effective method for selecting covariates in epidemiological analyses [21]. The final minimal adjustment set for COPD (Model 1) included socioeconomic status, smoking, sex, age, race, and BMI (Figure S1). The minimal adjustment set for asthma (Model 2) included the same variables for COPD, with the addition of family history of asthma (Figure S2). Model 2 covariates were also used to analyze the composite outcome of any obstructive airway disease. All analyses controlled for the corresponding NHANES cycles and the natural logarithm transformation of urinary creatinine values to adjust glyphosate concentrations for urinary dilution.

Subgroup analyses, stratified by age, were conducted for adults and youths. Analyses for adults included three age groups: (1) 20–39 (younger adults), (2) 40–59 (middle-aged), and (3) 60 and older (seniors). The age groups for the youths followed the Munich Age Classification System (MACS), which included: (1) 3–6 (early childhood), (2) 7–12 (middle childhood), and 13–19 (adolescence) [22]. Analysis for adults was conducted among individuals aged 20 years and above, in accordance with the classification adopted by NHANES. Because COPD data were available only for participants in this age group, it was necessary to adhere to NHANES criteria when defining the composite variable for any obstructive airway disease, thereby ensuring that all included participants could be appropriately analyzed.

In a sensitivity analysis, we examined the association between urine glyphosate measured at the time of data collection and time-bound outcomes to partially address temporality issues. Specifically, asthma attacks and emergency visits for asthma in the past year were assessed.

Following updated statistical interpretation guidance [23], both *p*-values and 95% uncertainty intervals (UIs) were reported. A *p* < 0.05 indicated statistical divergence, whereas *p* ≥ 0.05 indicated non-divergence (consistency with the tested hypothesis). Point estimates with 95% UIs represented the plausible range of population effects. Analyses were conducted using Stata version 18.5 (StataCorp LLC, College Station, TX, USA) [24]; model fit was assessed with McFadden’s R^2^, and specification with the link test.

## 3. Results

### 3.1. Study Population and Demographics

**Figure 1:**
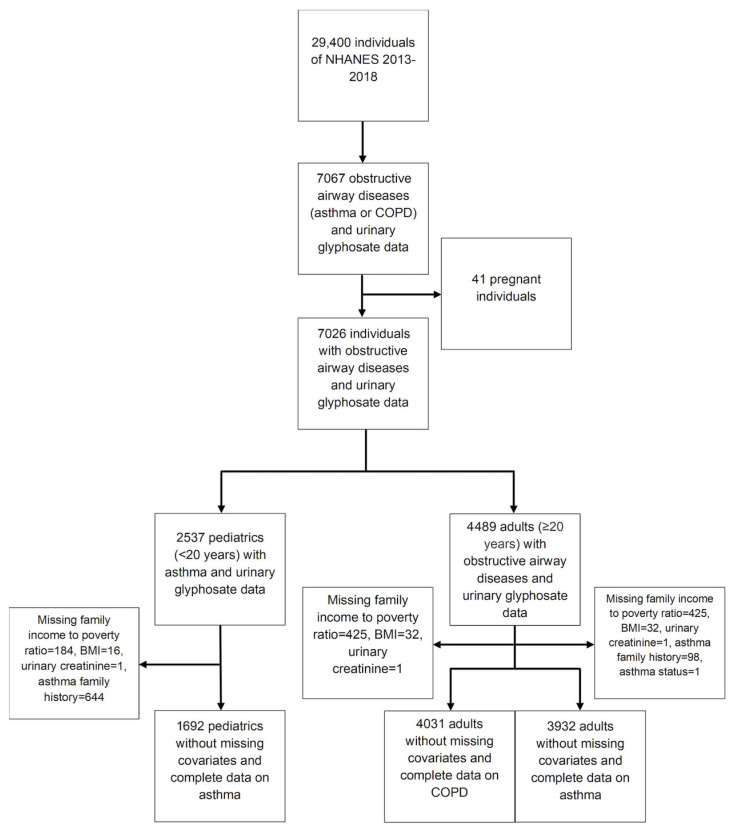
Flow diagram of participant selection for the study (NHANES 2013–18) with eligibility criteria and final sample size. BMI: body mass index; COPD: chronic obstructive pulmonary disease.

In total, 5723 participants were included (N = 4031 adults and N = 1692 youths) (Figure 1).

Around 20% of adults (N = 798; Table 1) had an obstructive airway disease. Of those, around 74% (N = 590) and 47.2% (N = 377) had COPD and asthma, respectively.

Statistical tests to assess group differences were the independent samples *t*-tests or Mann–Whitney U tests, depending on distribution. Categorical variables were compared using chi-squared or Fisher’s exact tests, as appropriate.

Among youths, around 20.4% had asthma (N = 346; Table 2).

Statistical tests to assess group differences were the independent samples *t*-tests or Mann–Whitney U tests, depending on distribution.

Categorical variables were compared using chi-squared or Fisher’s exact tests, as appropriate.

Participants’ characteristics for adults are presented in Table 1. The prevalence of airway diseases was higher in females compared to males (55.3% vs. 44.7%, *p* = 0.004). Most participants had a BMI of 30 kg/m^2^ or higher, including 38.5% in the non-airway disease group and 59.2% in the obstructive airway disease group (*p* < 0.001). The mean urine glyphosate level was comparable between those with and without obstructive airway disease, which were 0.51 ng/mL (SD = 0.56) and 0.49 ng/mL (SD = 0.58), respectively (*p* = 0.33). Individuals with obstructive airway diseases were more likely to fall within the higher glyphosate tertiles (*p* < 0.021). Participants’ characteristics and group differences by individual airway disease status (asthma or COPD) are presented in Tables S1 and S2, respectively.

In youths (Table 2), asthma was more prevalent in males (56.9%) than in females (43.1%; *p* = 0.096). Mean BMI was higher among those with asthma (22.7 ± 6.2 vs. 21.4 ± 5.9; *p* < 0.001), while mean urinary glyphosate levels were comparable (0.63 ± 0.64 vs. 0.61 ± 0.65 ng/mL; *p* = 0.594). Glyphosate tertile cut-offs by disease category are reported in Supplementary Tables S3 and S4.

### 3.2. The Relationship Between Urine Glyphosate and Obstructive Airway Diseases in Adults

Table 3 shows independent adjusted associations between urine glyphosate tertiles and obstructive airway diseases in adults. Participants in higher glyphosate tertiles had greater odds of obstructive airway diseases, with those in the third tertile showing 29% increased odds (95% UI: 1.02, 1.62; *p* = 0.03). Adults in the third tertile had 32% higher odds of asthma (95% UI: 1.02, 1.73; *p* = 0.04) compared to those in the first tertile, with statistical divergence. Participants in the second and third tertiles experienced higher odds of COPD; however, estimates were statistically nondivergent (*p* > 0.05).

### 3.3. The Relationship Between Urine Glyphosate and Asthma in Youths

In youths (Table 4), glyphosate tertiles showed weak, non-divergent associations with asthma. Compared to the first tertile, participants in the second and third tertiles had 3% decreased asthma odds (95% UI: 0.69, 1.36; *p* = 0.85) and 8% increased asthma odds (95% UI: 0.74, 1.58; *p* = 0.69), respectively.

### 3.4. Subgroup Analyses by Age

Potential associations were observed in adults aged 20–39; those in the second tertile had a twofold increase in the odds of COPD as compared to the first tertile (aOR = 2.1, 95% UI: 0.95, 4.65, *p* = 0.07). Meanwhile, the association between glyphosate and asthma was stronger in middle-aged adults, whereby those in the third tertile had 1.52 times the odds of asthma compared to the first tertile, although the UI included the null value (95% UI: 0.98, 2.35, *p* = 0.06). No significant dose–response relationships were observed in patients with COPD or asthma within individual age subgroups (Table 5).

In youths, increased odds of asthma were observed only in the third tertile among those in middle childhood (7–12 years), and in both the second and third tertiles among adolescents (13–19 years). There was no consistent dose–response trend across exposure tertiles. Conversely, younger children (3–6 years) demonstrate lower odds of asthma at both the second and third glyphosate tertiles (Table 5).

### 3.5. Results of the Sensitivity Analyses by Time-Bound Outcomes

Table 6 potentially shows an increasing trend in the odds of asthma events in the past year with higher glyphosate tertiles in adults, suggesting a potential dose–response association. Specifically, adult participants with urine glyphosate concentration at the second tertile had a 40% increase in the odds of requiring asthma emergency visits in the past year compared to those in the first tertile (95% UI:0.61, 3.21, *p* = 0.43). The increase in odds was higher in participants in the third tertile (Table 6). Similar trends were observed for past-year asthma attacks. Adults in the second and third tertiles had 60% (95% UI:0.87, 2.95, *p* = 0.13) and 75% (95% UI:0.92, 3.36, *p* = 0.09) higher odds of past-year asthma attacks, respectively, compared to those in the first tertile. However, it should be noted that the sensitivity analyses results in adults had weak evidence for statistical divergence.

Inconsistent trends were observed in the odds of past-year asthma events among youths (Table 6). Only participants with glyphosate concentrations at the second tertile had 54% higher odds of past-year asthma-related emergency visits (95% UI:0.55, 4.33, *p* = 0.41). Contrastingly, exposure levels in the lowest and highest tertiles were not significantly associated with past-year asthma events, with the highest tertile showing a slight, non-divergent decrease in odds (Table 6). This non-linear pattern suggests that the relationship between glyphosate exposure and time-bound outcomes in youths may not follow a straightforward dose–response trend.

## 4. Discussion

Using NHANES data from 2013 to 2018, we found that higher urine glyphosate concentrations could potentially be associated with increased odds of obstructive airway diseases, with effects seemingly more pronounced among younger and middle-aged adults, as well as adolescents. Our findings suggest a potential dose–response relationship between glyphosate and airway diseases in adults. Adults in higher tertiles of glyphosate exposure exhibited sequentially increasing odds of developing COPD and asthma. Strong dose–response patterns were also observed between glyphosate and past-year asthma-related events, suggesting a potential temporal association between recent exposure and respiratory outcomes. However, results on potential dose–response associations between glyphosate and asthma in youths were inconclusive.

Our findings on COPD differ from a recent study that also examined these associations using NHANES data. Shi et al. (2025) showed more than twofold increase in the odds of COPD with the third urinary glyphosate tertile (adjusted OR (Model 4) = 2.16; 95% UI: 1.11–4.2, *p* = 0.026), which was statistically divergent [15]. However, our study examined this relationship across a broader age range, including younger adults (20–39), and accounted for urinary creatinine in the analysis to provide a standardized assessment of glyphosate concentrations. In contrast, Shi et al. (2025) evaluated these associations in older individuals (≥40 years) and adjusted for chronic kidney disease [15], an approach that addresses biological differences in renal function but does not correct for urine dilution. This distinction may have biased their exposure estimates away from the null, potentially overestimating associations. This could also explain the weaker associations observed among older adults in our subgroup analysis.

Similar to our study, Li et al. (2023) included adults ≥ 20 years and adjusted for urinary creatinine to examine associations between glyphosate exposure and COPD [8]. However, unlike our findings, their results showed no associations. Notably, Li et al. (2023) analyzed data from two NHANES cycles (2013–2014 and 2015–2016) and applied relatively broad criteria for classifying COPD, which may have contributed to the null findings [8]. Neither of the two prior studies explored associations between glyphosate exposure and asthma in adults nor assessed these relationships in youths. Our study is therefore the first to present evidence linking glyphosate with a broader spectrum of airway diseases across multiple age groups.

The biological plausibility of our findings is supported by emerging mechanistic evidence [25,26,27]. For instance, air containing glyphosate may trigger airway inflammation through the IL-33 pathway, leading to the production of cytokines including IL-5 and IL-13, which are central to the pathophysiology of asthma [10]. In vitro studies documented mitochondrial malfunction and oxidative stress in human cells exposed to pesticides containing glyphosate [11]. In vivo murine models have demonstrated that continuous glyphosate exposure leads to lung inflammation through upregulation of adhesion molecules, including ICAM-1 and VCAM-1, which are known to facilitate the recruitment of immune cells and airway remodeling in chronic respiratory conditions [12].

Our findings align with a growing body of research linking glyphosate to adverse effects on pulmonary and systemic health [8,28,29]. One of the strengths of this study is the utilization of a large dataset (NHANES) that includes multiple age groups from both the adult and youth populations, enabling us to assess associations across a broad age range. Although our outcomes were self-reported, potentially subjecting analyses to risk of misclassification bias, disease classification was derived from a common population source and incorporated multiple indicators, including medication history and age of disease onset. The lack of statistical divergence, for instance, may reflect reliance on self-reported diagnoses rather than objective lung function measurements. On the other hand, our analyses controlled for urinary creatinine to account for variations in urine dilution, thereby improving the accuracy of urinary glyphosate measurements. Additionally, although the number of covariates included in our analyses was smaller than in the prior studies, variable selection was informed by a thorough literature review, guided by DAG modeling to minimize residual confounding and overadjustment.

Furthermore, to provide additional context regarding temporality, we examined time-bound outcomes, including past-year self-reported emergency visits, to capture recent health events potentially associated with current glyphosate exposure levels. However, the cross-sectional design limits causal inference. Glyphosate exposure was assessed at a single time point, which may not reflect cumulative or long-term exposure patterns. Finally, certain contextual factors, such as the extent of occupational exposure or regional variations in pesticide applications, could not be assessed due to insufficient data. Future prospective, longitudinal studies should be conducted to explore the temporal and chronic effects of glyphosate exposure.

Mechanistic studies should be prioritized to explore biological processes linking glyphosate to airway inflammation. Additionally, integrating air sample data with environmental exposure maps could improve exposure assessment in future research.

## 5. Conclusions

This study identified increased odds of obstructive respiratory diseases with higher glyphosate exposure across both adult and youth samples in NHANES. These associations appeared more pronounced among younger and middle-aged adults, as well as adolescents. Potential dose–response associations between glyphosate exposure and airway diseases were further exhibited among adults, particularly with recent asthma-related events. Our findings suggest the need for timely surveillance and preventative strategies, particularly in areas at risk of elevated glyphosate exposure. Future research should focus on longitudinal studies to explore causal pathways and cumulative exposure effects.

## Figures and Tables

**Table 1:** Participants’ characteristics in the adult population across three NHANES cycles (2013–2018) by obstructive airway diseases (N = 4031).

Characteristic	Category	No Airway Disease	Airway Disease	*p*-Value
Total N (%)		3233 (80.2)	798 (19.8)	
Age Category, n (%)	20–39 (Younger Adults)	1066 (33.0)	224 (28.1)	0.001
	40–59 (Middle Aged)	1111 (34.4)	261 (32.7)	
	≥60 (Seniors)	1056 (32.7)	313 (39.2)	
Sex, n (%)	Males	1629 (50.4)	357 (44.7)	0.004
	Females	1604 (49.6)	441 (55.3)	
Ethnicity, n (%)	White	1235 (38.2)	388 (48.)	<0.001
	Black	644 (19.9)	167 (20.9)	
	Asian	420 (13.0)	41 (5.1)	
	Hispanic	820 (25.4)	149 (18.7)	
	Other Non-Hispanic	420 (13.0)	41 (5.1)	
Family Income to Poverty Ratio, n (%)	≥1	2589 (80.1)	618 (77.4)	0.098
	<1	644 (19.9)	180 (22.6)	
Smoke Exposure, n (%)	No Smoke Exposure	1401 (43.3)	238 (29.8%)	<0.001
	Secondhand Smoking	405 (12.5)	103 (12.9)	
	Active Smoking	594 (18.4)	193 (24.2)	
	Both Active and Passive Smoking	833 (25.8)	264 (33.1)	
Employment Status, n (%)	Unemployed	1256 (38.8)	432 (54.1)	<0.001
	Employed	1977 (61.2)	366 (45.9)	
NHANES Cycle, n (%)	2013–2014	1212 (37.5)	274 (34.3)	0.105
	2015–2016	1037 (32.1)	252 (31.6)	
	2017–2018	984 (30.4)	272 (34.1)	
Mean Body Mass Index (SD)		29.3 (7.0)	31.0 (8.3)	<0.001
Body Mass Index Category, n (%)	Underweight, <18.5	44 (1.4)	21 (2.6)	<0.001
	Normal, 18.5 to <25	890 (27.5)	167 (20.9)	
	Overweight, 25 to <30	1053 (32.6)	217 (27.2)	
	Obese, ≥30	1246 (38.5)	393 (49.2)	
Chronic Kidney Disease, n (%)	No	2785 (88.0)	648 (84.3)	0.005
	Yes	379 (12.0)	121 (15.7)	
Family History of Asthma, n (%)	No	2603 (82.3)	485 (63.1)	<0.001
	Yes	561 (17.7)	284 (36.9)	
Mean Urinary Glyphosate (SD)		0.49 (0.58)	0.51 (0.5)	0.33
Glyphosate Tertile, n (%)	1	1101 (34.1)	233 (29.2)	0.021
	2	1080 (33.4)	274 (34.3)	
	3	1052 (32.5)	291 (36.5)	

SD: standard deviation.

**Table 2:** Participants’ characteristics in the youth population across three NHANES cycles (2013–2018) by asthma status (N = 1692).

Characteristic	Category	No Asthma	Asthma	*p*-Value
Total N (%)		1346 (79.6)	346 (20.4)	
Mean Age (SD), years		12.0 (4.0)	12.3 (4.0)	0.004
Age Category, n (%)	3–6 (Early childhood)	120 (8.9)	24 (6.9)	0.478
	7–12 (Middle childhood)	636 (47.3)	164 (47.4)	
	13–19 (Adolescence)	590 (43.8)	158 (45.7)	
Sex, n (%)	Males	699 (51.9)	197 (56.9)	0.096
	Females	647 (48.1)	149 (43.1)	
Ethnicity, n (%)	Asian	129 (9.6)	21 (6.1)	0.015
	White	402 (29.9)	99 (28.6)	
	Black	273 (20.3)	97 (28.0)	
	Hispanic	448 (33.3)	109 (31.5)	
	Other non-Hispanic	94 (7.0)	20 (5.8)	
Family Income to Poverty Ratio, n (%)	≥1	900 (66.9)	252 (72.8)	0.034
	<1	446 (33.1)	94 (27.2)	
Smoke Exposure, n (%)	No smoke exposure	1243 (92.3)	312 (90.2)	0.552
	Secondhand smoking	41 (3.0)	15 (4.3)	
	Active smoking	14 (1.0)	5 (1.4)	
	Both active and passive smoking	48 (3.6)	14 (4.0)	
Employment Status, n (%)	Employed	210 (65.2)	66 (67.3)	0.697
	Unemployed	112 (34.8)	32 (32.7)	
NHANES Cycle, n (%)	2013–2014	502 (37.3)	140 (40.5)	0.552
	2015–2016	459 (34.1)	111 (32.1)	
	2017–2018	385 (28.6)	95 (27.5)	
Mean Body Mass Index (SD), kg/m^3^		21.4 (5.9)	22.7 (6.2)	<0.001
Body Mass Index Category, n (%)	Underweight, <18.5	500 (37.1)	96 (27.7)	0.002
	Normal, 18.5 to <25	551 (40.9)	146 (42.2)	
	Overweight, 25 to <30	179 (13.3)	61 (17.6)	
	Obese, ≥30	116 (8.6)	43 (12.4)	
Chronic Kidney Disease, n (%)	No	1176 (87.4)	313 (90.5)	0.114
	Yes	170 (12.6)	33 (9.5)	
Family History of Asthma, n (%)	No	973 (72.3)	137 (39.6)	<0.001
	Yes	373 (27.7)	209 (60.4)	
Mean Urinary Glyphosate (SD)		0.61 (0.65)	0.63 (0.64)	0.594
Glyphosate Tertile, n (%)	1	400 (29.7)	97 (28.0)	0.481
	2	487 (36.2)	119 (34.4)	
	3	459 (34.1)	130 (37.6)	

SD: standard deviation.

**Table 3:** Associations between urinary glyphosate tertiles and obstructive airway diseases among adults.

	Tertiles	aOR	95% UI	*p*-Value
COPD * N = 4031				
	1	1 (base)		
	2	1.17	0.87, 1.57	0.31
	3	1.19	0.87, 1.65	0.28
Asthma ** N = 3932				
	1	1 (base)		
	2	1.16	0.91, 1.48	0.24
	3	1.32	1.02, 1.73	0.04
Any obstructive airway disease (asthma and/or COPD) ** N = 3933				
	1	1 (base)		
	2	1.17	0.94, 1.46	0.15
	3	1.29	1.02, 1.62	0.03

* Model 1: adjusted for age, sex, race, smoke exposure, family income to poverty ratio, NHANES cycle, urine creatinine, and BMI. ** Model 2: adjusted for model 1 covariates in addition to family history of asthma. Model 1 (COPD): goodness of fit pseudo R^2^ = 0.130; link test hat-squared *p* = 0.841. Model 2 (asthma): goodness of fit pseudo R^2^ = 0.073; link test hat-squared *p* = 0.123. Model 2 (any obstructive airway diseases, asthma and/or COPD): goodness of fit pseudo R^2^ = 0.072; link test hat-squared *p* = 0.680. Statistical divergence was set at *p*-value < 0.05. aOR: adjusted odds ratio; 95% UI: 95% uncertainty interval; COPD: chronic obstructive pulmonary disease.

**Table 4:** Association between urinary glyphosate and asthma among the youth.

	Tertiles	aOR	95% UI	*p*-Value
Asthma N = 1692				
	1	1 (base)		
	2	0.97	0.69, 1.36	0.85
	3	1.08	0.74, 1.58	0.69

Model-adjusted covariates include age, sex, race, smoke exposure, family income-to-poverty ratio, NHANES cycle, urine creatinine, BMI, and family history of asthma. Goodness of fit pseudo R^2^ = 0.091; link test hat-squared *p* = 0.154. Significance level was set at *p*-value < 0.05. aOR: adjusted odds ratio; 95% UI: 95% uncertainty interval; UI: uncertainty interval.

**Table 5:** Subgroup analysis by age in adults and youths.

Age Category	Tertiles	aOR	95% UI	*p*-Value
COPD *				
20–39 (Younger Adults) N = 1136	1	1 (base)		
	2	2.10	0.95, 4.65	0.07
	3	1.53	0.62, 3.79	0.36
40–59 (Middle Aged) N = 1372	1	1 (base)		
	2	0.92	0.55, 1.54	0.76
	3	1.18	0.68, 2.05	0.56
≥60 (Seniors) N = 1369	1	1 (base)		
	2	1.06	0.70, 1.60	0.78
	3	1.03	0.66, 1.60	0.91
Asthma **				
3–6 (Early Childhood) N = 767	1	1 (base)		
	2	0.97	0.54, 1.74	0.92
	3	0.74	0.38, 1.44	0.38
7–12 (Middle Childhood) N = 805	1	1 (base)		
	2	0.71	0.43, 1.17	0.18
	3	1.06	0.62, 1.80	0.83
13–19 (Adolescent) N = 759	1	1 (base)		
	2	1.33	0.83, 2.15	0.24
	3	1.20	0.69, 2.07	0.52
20–39 (Younger Adults) N = 1289	1	1 (base)		
	2	1.05	0.70, 1.57	0.83
	3	1.10	0.70, 1.71	0.69
40–59 (Middle Aged) N = 1372	1	1 (base)		
	2	1.19	0.79, 1.79	0.39
	3	1.52	0.98, 2.35	0.06
≥60 (Senior) N = 1369	1	1 (base)		
	2	1.18	0.77, 1.80	0.45
	3	1.25	0.79, 1.96	0.34

* Model 1: adjusted for age, sex, race, smoke exposure, family income to poverty ratio, NHANES cycle, urine creatinine, and BMI. ** Model 2: adjusted for model 1 covariates in addition to family history of asthma. Significance level was set at *p*-value < 0.05. aOR: adjusted odds ratio; 95% UI: 95% uncertainty interval; COPD: chronic obstructive pulmonary disease.

**Table 6:** Associations between urinary glyphosate and asthma events in the past year among adults and youths (sensitivity analysis).

	Tertiles	aOR	95% UI	*p*-Value
**Adults’ sensitivity analysis**				
**Asthma emergency visits** N = 345				
	1	1 (base)		
	2	1.40	0.61, 3.21	0.43
	3	1.58	0.66, 3.82	0.31
**Asthma attacks** N = 345				
	1	1 (base)		
	2	1.60	0.87, 2.95	0.13
	3	1.75	0.92, 3.36	0.09
**Youths’ sensitivity analysis**				
**Asthma emergency visits** N = 204				
	1	1 (base)		
	2	1.54	0.55, 4.33	0.41
	3	0.49	0.15, 1.59	0.24
**Asthma attacks** N = 209				
	1	1 (base)		
	2	1.01	0.44, 2.30	0.99
	3	0.45	0.18, 1.13	0.09

Models adjusted for covariates include age, sex, race, smoke exposure, family income to poverty ratio, NHANES cycle, urine creatinine, BMI, and family history of asthma. Adults’ emergency visits: goodness of fit pseudo R^2^ = 0.083; goodness of link *p* = 0.912. Adults’ asthma attacks: goodness of fit pseudo R^2^ = 0.120; goodness of link *p* = 0.507. Youths’ emergency visits: goodness of fit pseudo R^2^ = 0.154; goodness of link *p* = 0.984. Youths’ asthma attacks: goodness of fit pseudo R^2^ = 0.098; goodness of link *p* = 0.598. Significance level was set at *p*-value < 0.05. aOR: adjusted odds ratio; 95% UI: 95% uncertainty interval; UI: uncertainty interval.

## Data Availability

The datasets generated and/or analyzed during the current study are publicly available in the NHANES repository, https://www.cdc.gov/nchs/nhanes/index.html accessed on 26 February 2025.

## Supplementary Materials

The following supporting information can be downloaded at: https://www.mdpi.com/article/10.3390/ijerph23040442/s1, Figure S1: The Directed Acyclic Graph (DAG) showing the hypothesized relationship between creatine-adjusted urinary glyphosate levels and COPD, adjusted for potential confounders; Figure S2: The Directed Acyclic Graph (DAG) showing the hypothesized relationship between creatine-adjusted urinary glyphosate levels and Asthma, adjusted for potential confounders; Table S1: Demographic and health characteristics of the adult population in the US across three NHANES cycles (2013–2018) by asthma status, N = 3932; Table S2: Demographic and health characteristics of the adult population in the US across three NHANES cycles (2013–2018) by COPD status, N = 4031; Table S3: Mean Urine Glyphosate in each Tertile by Disease Category in Adults; Table S4: Mean Urine Glyphosate in each Tertile by Asthma Status in Pediatrics.
