# Supplementary material for: Urinary Glyphosate Exposure and Risk of Obstructive Airway Diseases in Youth and Adults: Population-Based Evidence from U.S. Biomonitoring Data
Source: Int J Environ Res Public Health. 2026 Mar 31;23(4):442. doi: 10.3390/ijerph23040442 (PMC13116281; doi:10.3390/ijerph23040442)
Supplement: Supplementary file 1 [file ijerph-23-00442-s001.zip › ijerph-4179231-supplementary.pdf]

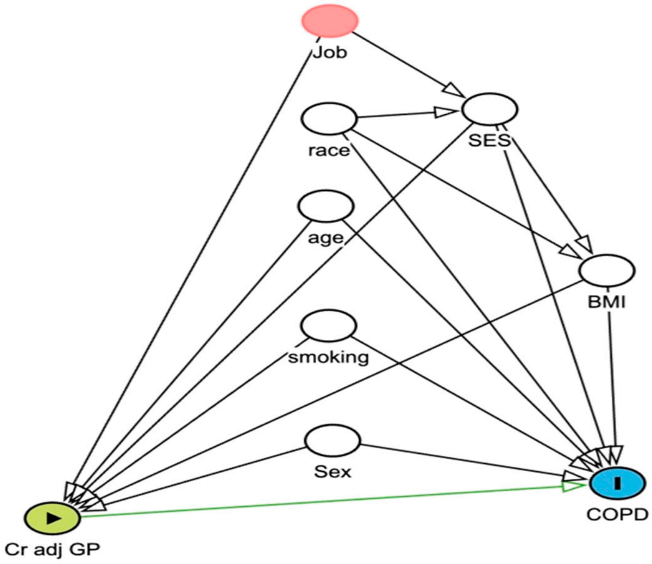

Figure S1. The Directed Acyclic Graph (DAG) showing the hypothesized relationship between creatine-adjusted urinary glyphosate levels and COPD, adjusted for potential confounders.

This DAG identifies the minimum set of confounders included in Model 1. White circles depict adjusted variables. **BMI:** body mass index; **COPD:** chronic obstructive pulmonary disease; **Cr adj GP:** creatinine adjusted glyphosate; **SES:** socioeconomic status represented by the family income-to-poverty ratio.

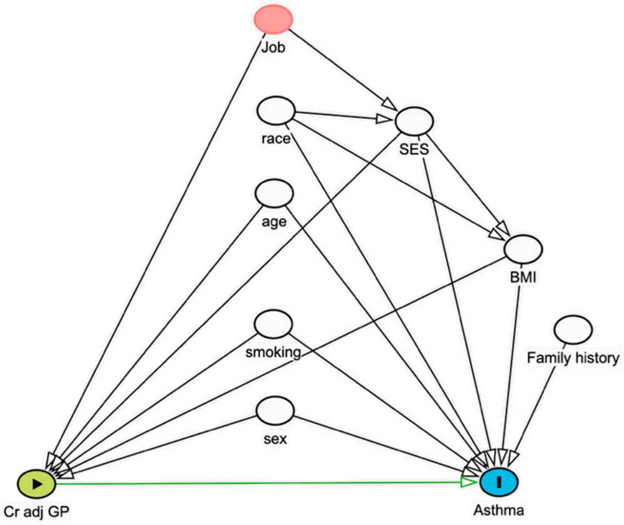

Figure S2. The Directed Acyclic Graph (DAG) showing the hypothesized relationship between creatine-adjusted urinary glyphosate levels and Asthma, adjusted for potential confounders.

This DAG identifies the minimum set of confounders included in Model 2. White circles depict adjusted variables. White circles depict adjusted variables. **BMI:** body mass index; **Cr adj GP:** creatinine adjusted glyphosate; **SES:** socioeconomic status represented by the family income-to-poverty ratio.

Table S1. Demographic and health characteristics of the adult population in the US across three NHANES cycles (2013-2018) by asthma status, N=3,932.

Characteristic	Category	No Asthma	Asthma	p-value
Total N (%)		3,366 (85.6)	566 (14.4)	
Mean Age (SD)		50.0 (17.5)	49.0 (17.4)	0.207
Age Category, n (%)	20-39 (Adults)	1,072 (31.8)	192 (33.9)	0.584
	40-59 (Middle Aged)	1,143 (34.0)	190 (33.6)	
	≥60 (Senior)	1,151 (34.2)	184 (32.5)	
Sex, n (%)	Males	1,682 (50.0)	243 (42.9)	0.002
	Females	1,684 (50.0)	323 (57.1)	
Ethnicity, n (%)	Asian	413 (12.3)	33 (5.8)	<0.001
	White	1,322 (39.3)	254 (44.9)	
	Black	667 (19.8)	127 (22.4)	
	Hispanic	840 (25.0)	114 (20.1)	
	Other Non-Hispanic	124 (3.7)	38 (6.7)	
Family Income to Poverty Ratio, n (%)	≥1	2,700 (80.2)	440 (77.7)	0.174
	<1	666 (19.8)	126 (22.3)	
Smoke Exposure, n (%)	No smoke exposure	1,411 (41.9)	190 (33.6)	<0.001

	Secondhand smoking	407 (12.1)	89 (15.7)	
	Active smoking	637 (18.9)	126 (22.3)	
	Both active and passive smoking	911 (27.1)	161 (28.4)	
Employment Status, n (%)	Unemployed	1,364 (40.5)	279 (49.3)	<0.001
	Employed	2,002 (59.5)	287 (50.7)	
NHANES Cycle, n (%)	2013-2014	1,256 (37.3)	198 (35.0)	0.567
	2015-2016	1,062 (31.6)	186 (32.9)	
	2017-2018	1,048 (31.1)	182 (32.2)	
Mean Body Mass Index (SD)		29.3 (7.1)	31.3 (8.4)	<0.001
Body Mass Index Category, n (%)	Underweight, <18.5	53 (1.6)	11 (1.9)	<0.001
	Normal, 18.5 to <25	916 (27.2)	116 (20.5)	
	Overweight, 25 to <30	1,083 (32.2)	156 (27.6)	
	Obese, ≥30	1,314 (39.0)	283 (50.0)	
Chronic Kidney Disease, n (%)	No	2,947 (87.6)	485 (85.7)	0.218
	Yes	419 (12.4)	81 (14.3)	
Family History of Asthma, n (%)	No	2,766 (82.2)	321 (56.7)	<0.001
	Yes	600 (17.8)	245 (43.3)	
Mean Urinary Glyphosate (SD)		0.49 (0.57)	0.50 (0.57)	0.505
Glyphosate Tertile, n (%)	1	1,135 (33.7)	168 (29.7)	0.102
	2	1,130 (33.6)	191 (33.7)	
	3	1,101 (32.7)	207 (36.6)	

SD: standard deviation.

Statistical tests to assess group differences were the independent samples t-tests or Mann–Whitney U tests, depending on distribution. Categorical variables were compared using chi-squared or Fisher's exact tests, as appropriate.

Table S2. Demographic and health characteristics of the adult population in the US across three NHANES cycles (2013-2018) by COPD status, N=4,031.

Characteristic	Category	No COPD	COPD	p-value
Total N (%)		3,654 (90.6)	377 (9.4)	
Mean Age (SD)		48.9 (17.4)	58.9 (15.7)	<0.001
Age Category, n (%)	20-39 (Adults)	1,241 (34.0)	49 (13.0)	<0.001
	40-59 (Middle Aged)	1,250 (34.2)	122 (32.4)	
	≥60 (Senior)	1,163 (31.8)	206 (54.6)	
Sex, n (%)	Males	1,821 (49.8)	165 (43.8)	0.025
	Females	1,833 (50.2)	212 (56.2)	
Ethnicity, n (%)	Asian	450 (12.3)	11 (2.9)	<0.001
	White	1,417 (38.8)	206 (54.6)	
	Black	744 (20.4)	67 (17.8)	
	Hispanic	908 (24.8)	61 (16.2)	
	Other Non-Hispanic	135 (3.7)	32 (8.5)	
Family Income to Poverty Ratio, n (%)	≥1	2,920 (79.9)	287 (76.1)	0.083
	<1	734 (20.1)	90 (23.9)	

Smoke Exposure, n (%)	No smoke exposure	1,566 (42.9)	73 (19.4)	<0.001
	Secondhand smoking	479 (13.1)	29 (7.7)	
	Active smoking	684 (18.7)	103 (27.3)	
	Both active and passive smoking	925 (25.3)	172 (45.6)	
Employment Status, n (%)	Unemployed	1,428 (39.1)	260 (69.0)	<0.001
	Employed	2,226 (60.9)	117 (31.0)	
NHANES Cycle, n (%)	2013-2014	1,368 (37.4)	118 (31.3)	0.013
	2015-2016	1,171 (32.0)	118 (31.3)	
	2017-2018	1,115 (30.5)	141 (37.4)	
Mean Body Mass Index (SD)		29.4 (7.2)	31.0 (8.3)	<0.001
Body Mass Index Category, n (%)	Underweight, <18.5	50 (1.4)	15 (4.0)	<0.001
	Normal, 18.5 to <25	984 (26.9)	73 (19.4)	
	Overweight, 25 to <30	1,171 (32.0)	99 (26.3)	
	Obese, ≥30	1,449 (39.7)	190 (50.4)	
Chronic Kidney Disease, n (%)	No	3,206 (87.7)	308 (81.7)	<0.001
	Yes	448 (12.3)	69 (18.3)	
Mean Urinary Glyphosate (SD)		0.49 (0.57)	0.51 (0.62)	0.453
Glyphosate Tertile, n (%)	1	1,225 (33.5)	108 (28.6)	0.153
	2	1,221 (33.4)	133 (35.3)	
	3	1,207 (33.0)	136 (36.1)	

SD: standard deviation.

Statistical tests to assess group differences were the independent samples t-tests or Mann–Whitney U tests, depending on distribution. Categorical variables were compared using chi-squared or Fisher's exact tests, as appropriate.

Table S3. Mean Urine Glyphosate in each Tertile by Disease Category in Adults.

Disease	Category	Glyphosate Tertile, Mean ng/mL (SD)		
		1	2	3
COPD*	No	0.13 (0.036)	0.33 (0.066)	1.01 (0.75)
	Yes	0.13 (0.039)	0.33 (0.067)	0.99 (0.82)
Asthma	No	0.13 (0.036)	0.33 (0.066)	1.01 (0.74)
	Yes	0.14 (0.037)	0.33 (0.066)	0.96 (0.74)
Any obstructive airway disease	No	0.13 (0.036)	0.33 (0.066)	1.02 (0.75)
	Yes	0.14 (0.038)	0.33 (0.065)	0.96 (0.69)

Glyphosate tertile cut-offs are 0.21 and 0.45 (minimum value= 0.071, maximum value= 8.21). *The first glyphosate cut-off is 0.22 with the same remaining values.

Table S4. Mean Urine Glyphosate in each Tertile by Asthma Status in Pediatrics.

Disease	Category	Glyphosate Tertile, Mean ng/mL (SD)		
		1	2	3

Asthma	No	0.16 (0.058)	0.42 (0.086)	1.20 (0.81)
	Yes	0.17 (0.052)	0.40 (0.090)	1.19 (0.74)

Glyphosate tertile cut-offs are 0.31 and 0.60 (minimum value= 0.071, maximum value= 8.13).
